# *“Fast but not so furious”*: A condensed step‐up dosing schedule of teclistamab for relapsed/refractory multiple myeloma

**DOI:** 10.1002/jha2.906

**Published:** 2024-07-08

**Authors:** Kevin C. Graf, James A Davis, Alyssa Cendagorta, Katelynn Granger, Kelly J. Gaffney, Kimberly Green, Brian T Hess, Hamza Hashmi

**Affiliations:** ^1^ Department of Medicine, Division of Hematology/Oncology Medical University of South Carolina Charleston South Carolina USA; ^2^ Department of Pharmacy Medical University of South Carolina Charleston South Carolina USA; ^3^ Department of Medicine Multiple Myeloma service Memorial Sloan Kettering Cancer Center New York New York USA

**Keywords:** immunotherapy, multiple myeloma, myeloma therapy

## Abstract

Teclistamab is a B‐cell maturation antigen (BCMA)‐directed bispecific T‐cell engager approved for relapsed‐refractory multiple myeloma (RRMM). Cytokine release syndrome (CRS) and Immune effector cell‐associated neurotoxicity syndrome (ICANS) are well‐documented treatment ‐related adverse events of teclistamab. The prescribing information recommends step‐up dosing on days 1, 4, and 7 with 48–72 h of inpatient observation after each dose to monitor for CRS. This leads to a more than weeklong hospital stay, adding to the cost of therapy, resource utilization, and patient inconvenience. Here, we present a single center retrospective analysis addressing the safety and utility of a condensed step‐up dosing schedule for teclistamab. All patients who were treated with teclistamab from November 2022 to August 2023 at the Medical University of South Carolina were included in the analysis. Patients received subcutaneous (SC) teclistamab with step‐up doses (0.06 and 0.3 mg/kg) separated by either 2 or 3 (48–72 h) before the administration of the first full (1.5 mg/kg) dose (days 1, 3, and 5 ‘condensed’ schedule or days 1, 4, and 7 ‘standard’ schedule, respectively). All patients were hospitalized for the two step‐up doses and first full dose of teclistamab and received pre‐medications prior to each dose. Patients could be discharged after a minimum of 24 h following the full dose, if they did not have any CRS or ICANS. Relevant data regarding incidence, severity, and onset of CRS was collected. Statistical analysis was completed to assess the probability of fever with the first full dose of teclistamab based on incidence of fever with previous doses. A total of 25 patients were included in the analysis. Twenty‐eight percent (7/25) of patients underwent the standard step up while the remaining 72% (18/25) underwent a condensed step up of teclistamab. More than half (53%, 13/25) of the patients experienced CRS during step up dosing. Grades 1 and 2 CRS occurred in 48% (12/25) and 4% (1/25) patients, respectively. Of the 13 patients that experienced CRS, 30% (4/13) fevered with the first dose, 84% (11/13) fevered with the second dose, and one patient developed fever after the third dose. The negative predictive value of being ‘fever free’ after doses 1 and 2 and remaining ‘fever free’ throughout hospitalization was 0.92. The median length of hospital stay among the 1, 3, and 5 step up group was 6 days (6–25) and 70% (14/20) of patients were discharged from the hospital within 7 days of treatment initiation. This report demonstrates the utility of a condensed step‐up schedule for teclistamab initiation. The schedule was found to be safe and reduced hospital length of stay. These results should prompt consideration of shorter hospital stays for patients who do not experience CRS and raise the possibility of outpatient administration with close observation.

## 1 INTRODUCTION

Patients with heavily pretreated relapsed/ refractory multiple myeloma (RRMM), refractory to all three major drug classes including immunomodulatory drugs (IMiDs), proteasome inhibitors (PI), and anti‐CD38 monoclonal antibodies (mAb) have poor prognoses with a median progression‐free survival (PFS) of 3–4 months and overall survival (OS) of 5.6–13 months [1, 2, 3]. Prior to the approval of B‐cell maturation antigen (BCMA)‐directed cellular immunotherapies including chimeric antigen receptor (CAR) T‐cell therapy, patients with triple and penta‐refractory disease had limited treatment options and constituted an unmet need in the therapeutic landscape of RRMM. More recently, bispecific antibodies have been incorporated into the treatment paradigm of RRMM after showing impressive outcomes in pivotal trials evaluating their safety and efficacy. In October 2022, teclistamab was the first bispecific agent to receive US Food and Drug Administration (USFDA) approval for RRMM based on the phase I/II MajesTEC‐1 study [4]. In a heavily pre‐treated patient population, with a median of five prior lines of therapy (LOT), teclistamab monotherapy yielded an overall response rate (ORR), and complete response or better (≥CR) rate of 63% and 39%, respectively. The median PFS was 11.3 months and the median OS was 18.3 months. Cytokine release syndrome (CRS) was observed in 72% (grade ≥3: 0.6%) and neurotoxicity in 14.5% (grade ≥3: 0%) of the patients, respectively. Given the reasonable safety and efficacy profiles of this emerging treatment modality, other BCMA and non‐BCMA‐directed bispecifics have also been approved for commercial use [5, 6].

Teclistamab functions as a bispecific antibody engineered to link CD3 expressed on the surface of T‐cells and BCMA expressed on the surface of plasma cells, leading to immunologic cell death of BCMA‐expressing myeloma cells [7]. Largely mediated by the release of interleukin 6 and interferon‐gamma, CRS can present as a complication of cellular immunotherapy with signs of fever, hemodynamic instability, and/ or hypoxia [8]. Given the incidence and severity of CRS, special attention has been placed in clinical trials on the strategic administration of this agent and guideline‐directed prevention and management of the toxicity. The current prescribing information recommends step‐up dosing on days 1, 4, and 7 with pre‐medications administered prior to each dose. Patients are to be observed for 48–72 h after each step‐up dose to monitor for CRS and neurotoxicity. Depending on institution‐specific guidelines, administration of teclistamab may entail a continuous hospitalization for the initiation and step‐up regimen or alternatively, outpatient administration with subsequent hospitalization as needed for monitoring or management of toxicities. If this recommended regimen were initiated and followed in an inpatient setting, patients would be hospitalized for a minimum of 9 days, adding to the cost of therapy, healthcare resource utilization, and patient inconvenience. Here, we present an academic center experience addressing the safety and utility of a condensed step‐up dosing schedule for inpatient administration of teclistamab.

## 2 METHODS

We performed a single‐center retrospective analysis of adult patients with RRMM who received teclistamab under a commercial FDA label between November 2022 and August 2023. Approval was obtained from the institutional review board, which granted a waiver of consent, and the study was conducted in accordance with the Declaration of Helsinki. At the time of data cutoff (August 15, 2023), all patients had received at least one full dose of teclistamab for the safety analysis.

Similar to the regulatory approval guidelines available in the package insert, patients received subcutaneous teclistamab in step‐up doses of 0.06 mg per kilogram and 0.3 mg per kilogram followed by a full dose of 1.5 mg per kilogram administered once weekly. However, step‐up doses were separated by 2–3 days (48‐72 h) and were completed 2–3 days (48‐72 h) before the administration of the first full teclistamab dose (days 1, 3, and 5 ‘condensed’ schedule or days 1, 4, and 7 ‘standard’ schedule, respectively). This modification in administration schedule was guided by a change in institutional protocol due to the growing experience with teclistamab associated CRS and its management. All patients were hospitalized for the two step‐up doses and first full dose of teclistamab and received oral pre‐medications with dexamethasone 20 mg, acetaminophen 650 mg, and diphenhydramine 50 mg prior to each dose. Patients were monitored for signs and symptoms of CRS every 4 h and absence of symptoms was required for a minimum of 24 h prior to administration of subsequent doses. In the absence of toxicities and after two step‐up doses and the first full dose of teclistamab, patients were discharged for outpatient administration of subsequent weekly doses. Teclistamab was continued weekly until the occurrence of disease progression, unacceptable toxicity, or death.

CRS and immune‐effector cell‐associated neurotoxicity syndrome (ICANS) were graded according to the American Society for Transplantation and Cellular Therapy criteria [9]. Management of CRS and ICANS as per institutional guidelines which dictate treatment with a combination of acetaminophen, dexamethasone, and/or tocilizumab in addition to any other general diagnostic and therapeutic interventions specific to symptoms. Relevant data regarding the incidence, onset, and severity of CRS were collected. The primary objective was to determine the incidence of CRS with the step‐up doses and assess whether it could be predictive of the overall incidence of CRS with the first full dose with either administration schedule (standard vs. condensed). This data was used to extrapolate a negative predictive value of remaining fever‐free with the third (full) dose of teclistamab after being fever‐free with the first two doses in the ramp‐up.

## 3 RESULTS

**FIGURE 1:**
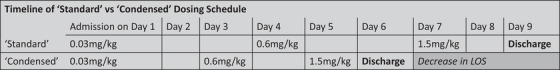
Step‐up dosing schedules (legend only). Example schedule representing admission to hospital and first dose on day 1 followed by inpatient observation throughout the entirety of treatment. Subsequent doses 48–72 h apart depending on which schedule patient received. Patients in the ‘condensed’ group could be discharged from the hospital 24 h after the final dose if no incidence of cytokine release syndrome (CRS).

A total of 25 patients were included in the analysis. The median patient age was 66 years (range 37–78). Thirteen were male (13/25, 52%) and 14 (56%) self‐identified as white. The median number of prior LOT was 5 (range 2–12). Forty‐four percent (11/25) had received prior BCMA‐directed therapy (Table 1). Among the 25 patients included in the report, seven (28%) patients received teclistamab according to the ‘standard’ ramp‐up dosing schedule on days 1, 4, and 7, while the remaining 18 (72%) patients received a ‘condensed’ ramp up schedule receiving teclistamab on days 1, 3, and 5 as outlined in Figure 1.

**TABLE 1:** Patient characteristics, toxicities, treatments, and length of stay.

Characteristics	Median (range) or *n*, (%)
Age	66 (37–78)
Male sex,	13 (52)
Race, non‐Hispanic White versus not	14 (56)
Prior lines of therapy	5 (2–12)
Triple class refractory	20 (80)
Penta class refractory	12 (48)
Extramedullary disease	13 (52)
High‐risk cytogenetics	9 (36)
Prior BCMA therapy	11 (44)
BM plasma cell > 50%	7 (54)***

Toxicities	*n*, (%)
CRS, any grade	13 (52)
CRS, grade 1	12 (48)
CRS, grade 2	1 (4)
ICANS, any grade	1 (4)

Treatment of CRS	*n*, (%)
Acetaminophen*	13 (52)
Dexamethasone**	12 (48)
Tocilizumab	3 (12)

Length of Hospital Stay (Days)	Median (range)
Days 1, 4, and 7 schedule (*n* = 5)	9 (8–11)
Days 1, 3, and 5 schedule (*n* = 20)	6 (6–25)

Abbreviations: BCMA, B‐cell maturation antigen; BM, bone marrow; CRS, cytokine release syndrome; ICANS, immune‐effector cell associated neurotoxicity syndrome; n, number.

* Indication specifically for CRS.

** Dexamethasone administration is given in addition to standard pretreatment for treatment.

*** Thirteen patients had BM biopsy data available.

Of the 25 patients who received teclistamab, 13 (52%) experienced CRS, all of which were grade 1 in intensity except for one grade 2 (4%). Among all patients who experienced CRS, 30% (4/13) fevered with the first dose while 84% (11/13) fevered with the second dose. Only one patient developed a fever with the third dose despite not experiencing fever with the prior two doses. The negative predictive value of being fever‐free throughout the teclistamab ramp‐up after being fever‐free with the first two step‐up doses was 0.92. All patients with CRS were managed with acetaminophen, 12 (92%) received dexamethasone, and three (23%) received tocilizumab. The median length of hospital stay for the standard schedule was 9 days (range 8–11). In comparison, the median length of stay in the condensed schedule was 6 days (range 6–25). Of all patients in the condensed schedule, 70% were discharged from the hospital within 7 days of treatment initiation.

## 4 DISCUSSION

As the use of T‐cell engager therapy increases, the evolution of therapy administration and treatment schedules is paramount to optimize patient care and experience. Prophylactic tocilizumab as presented by Nooka et al to facilitate outpatient administration of therapy, and center‐specific modifications to dosing schedules have allowed for variations in treatment with the hope for improvements in patient care [10]. There are several aspects of our condensed administration schedule of teclistamab that need to be highlighted to show its impact on the clinical management of patients with RRMM. While CRS was observed in nearly half the patients, all cases were low grade, easily managed with supportive care measures, and did not require transfer to the intensive care unit or lead to discontinuation of therapy. The incidence and severity of CRS were overall similar to what has been reported historically [4]. More importantly, none of the patients experienced late onset CRS or ICANS after hospital discharge. Patients who did not experience fever with the first two step‐up doses had a high probability of remaining fever‐free with the third/full dose of teclistamab, raising the possibility of an early discharge (24 h) after the second dose, further shortening the length of hospital stay. However, care needs to be exercised during patient selection. Specifically, risk factors for development of more severe CRS such as tumor burden need to be carefully considered when selecting patients for such intervention, as only a small proportion of patients had bone marrow plasma cell content of > 50% and other measures of disease burden where not accounted for. Strategies for appropriate patient selection may consist of clear inclusion criteria, precise implementation of appropriate premedication strategies, and close post‐discharge follow‐up. Early discharge may not be appropriate for patients with major comorbidities, limited caregiver support, or extensive disease burden. As late onset and recurrent CRS have been reported, patients should be counseled on how to monitor for CRS at home and when to seek help from their healthcare team [4].

This study has major implications for clinical practice both at academic centers and community practice‐based hospitals given the impact on the cost of hospital stay and healthcare resource utilization. Specifically, at our institution, the total cost of teclistamab therapy is approximately $2250/night. The acquisition cost of the drug itself makes up only a fraction of that cost and a majority is due to general hospital expenses of admission. Using these estimates a decrease in length of stay of 3 days could realize a $6750 decrease in total costs of admission. Broadly, this dosing schedule, if implemented at a departmental level with a theoretical number of 50 patients a year would equate to a total decrease in costs of $337,500. Hence, abbreviating the step‐up dosing schedule of teclistamab can be a highly cost‐effective strategy with substantial monetary savings. Additionally, the conservation of resources that comes with decreased length of stay can be a valuable metric.

Limitations of our analysis include it being a single‐center retrospective experience with monetary comments and predictions limited only to estimates in the United States. Additionally, this study includes a small number of patients treated over a relatively short period of time. However, outcomes with the incidence, timing, and severity of CRS as highlighted in our analysis are not significantly different from what has been reported previously. Due to the retrospective nature of the study, patients were sequentially assigned to the standard and condensed schedule. However, the choice of a condensed schedule was based on a change in institutional protocol and was not physician‐dependent, eliminating selection bias. While our data clearly indicates there is improvement in length of stay and associated costs with a condensed administration schedule, we did not collect data regarding patient satisfaction or quality of life, though anecdotally we do believe that shorter hospital stays lead to improved patient satisfaction for these patients.

## 5 CONCLUSION

This report demonstrates the utility of a condensed step‐up dosing schedule for teclistamab initiation. The schedule was found to be safe and reduced the length and cost of hospital stays. These results should prompt consideration of shorter hospital stays for patients who do not experience CRS with the first two step‐up doses and raise the possibility of an entirely outpatient administration with close observation.

## AUTHOR CONTRIBUTIONS

Kevin C. Graf and Hamza Hashmi wrote the manuscript. All authors reviewed the final manuscript before submission.

## CONFLICT OF INTEREST STATEMENT

Hamza Hashmi is on the advisory boards of Sanofi, BMS, and Janssen. The rest of the authors declare no conflict of interest.

## FUNDING INFORMATION

This research did not receive any specific grant from funding agencies in the public, commercial, or not‐for‐profit sectors.

## ETHICS STATEMENT

This study obtained Institutional Review Board approval, and the study was conducted according to the Declaration of Helsinki.

## PATIENT CONSENT STATEMENT

N/A due to the retrospective nature of the study, however, this study obtained Institutional Review Board approval, and the study was conducted according to the Declaration of Helsinki.

## CLINICAL TRIAL REGISTRATION

The authors have confirmed clinical trial registration is not needed for this submission.

## Data Availability

Data are available upon request.
